# The relationship between phylogenetic classification, virulence and antibiotic resistance of extraintestinal pathogenic *Escherichia coli* in İzmir province, Turkey

**DOI:** 10.7717/peerj.5470

**Published:** 2018-08-24

**Authors:** Elif Bozcal, Vahap Eldem, Sohret Aydemir, Mikael Skurnik

**Affiliations:** 1 Department of Biology, Basic and Industrial Microbiology Section, Faculty of Science, Istanbul University, Istanbul, Turkey; 2 Department of Biology, Zoology Section, Faculty of Science, Istanbul University, Istanbul, Turkey; 3 Department of Medical Microbiology, Faculty of Medicine, Ege University, İzmir, Turkey; 4 Department of Bacteriology and Immunology, Medicum and Research Programs Unit, Immunobiology, University of Helsinki, Helsinki, Finland

**Keywords:** *Escherichia coli*, 16S rRNA, antibiotic resistance, MLST, Virulence, ST131

## Abstract

**Background:**

Extraintestinal pathogenic *Escherichia coli* (ExPEC) is an important bacterium and responsible for many bloodstream infections, including urinary tract infections and even fatal bacteremia. The aim of this research was to investigate whether ExPEC strains isolated from Turkish blood cultures have a relationship between 16S rRNA based phylogenetic clusters and antibiotic resistance profiles, virulence factors or clonal lineages.

**Methods:**

Phenotypically identified ExPEC blood culture isolates (*n* = 104) were included in this study. The 16S rRNA partial sequence analysis was performed for genotypic identification of ExPEC isolates. Antibiotic susceptibility and Extended-Spectrum β-Lactamase testing of isolates were performed. Phylogenetic classification (A, B1, B2 and D), Multi Locus Sequence Typing analysis and virulence-associated genes were investigated.

**Results:**

Based on 16S rRNA partial sequence analysis, 97 out of 104 (93.26%) ExPEC isolates were confirmed as *E. coli*. Ampicillin (74.22%) and cefuroxime axetil (65.97%) resistances had the highest frequencies among the ExPEC isolates. In terms of phylogenetic classification of ExPEC, D (38.14%, 37/97) was the most prevalent group after A (29.89%, 29/97), B2 (20.61%, 20/97), and B1 (11.34%, 11/97). The sequence types of the 20 ExPEC isolates belonging to the B2 phylogenetic group were analyzed by Multi Locus Sequence Typing. Ten isolates out of 20 (50.0%) were identified as ST131. The other STs were ST95 (*n* = 1), ST14 (*n* = 1), ST10 (*n* = 1), ST69 (*n* = 1), ST1722 (*n* = 2), ST141 (*n* = 1), ST88 (*n* = 1), ST80 (*n* = 1), and ST998 (*n* = 1). Of the ST131 strains, six (60%, 6/10) represented serogroup O25. The most common virulence factor genes were serum resistance factor gene, *traT* (55.7%) aerobactin siderophore receptor and yersiniabactin encoding genes *iutA* (45.3%) and* fyuA* (50.5%), respectively. In addition, PAI (41.2%), *iroN* (23.7%), *hlyA* (15.4%), *kpsII* (13.4%),* ompT* (13.4%), *papG* (12.4%), *iss* (9.3%),* cnf1* (7.2%),* ibeA* (2.06%), and *sfaS* (2.06%) genes were present in the ExPEC isolates.

**Conclusion:**

The 16S rRNA-based phylogenetic relationship tree analysis showed that a large cluster was present among 97 ExPEC isolates along with related reference strains. There were 21 main clusters with 32 closely related subclusters. Based on our findings, different clonal lineages of ExPEC can display different antibiotic susceptibilities and virulence properties. We also concluded that virulence factors were not distributed depending on phylogenetic groups (A, B1, B2, and D). The ExPEC isolates belonging to the same phylogenetic group and sequence type could display different resistance and virulence characteristics.

## Introduction

*Escherichia coli* (*E. coli*) is one of the most thoroughly studied bacterium of the human intestinal system (Koga et al., 2014). Despite the occurrence of commensal strains in the intestinal microbiota, *E. coli* can cause to various infections not only in the intestinal system but also in the bloodstream. *E. coli* that can cause infections outside the intestinal system are designated as extra intestinal pathogenic *E. coli* (ExPEC) (Ananias & Yano, 2008; Fratamico et al., 2016). ExPEC can initiate systemic infections by causing sepsis or bacteremia that may disseminate bacteria to tissues and organs including the urinary tract, central nervous system, and deep surgical wound areas (Chouikha et al., 2006; Lloyd et al., 2009).

ExPEC has been divided into four groups (A, B1, B2, and D) according to the phylogenetic classification. Pathogenic groups of ExPEC strains generally belong to groups B2 and D. However, commensal strains that survive in the intestinal system, i.e., non-pathogenic strains, are generally included in groups A or B1 (Clermont, Bonacorsi & Bingen, 2000). In addition to the phylogenetic grouping, Multi-Locus Sequence Typing (MLST) of ExPEC is a typing method used to identify clinically significant ExPEC ST lineages where strains fall into certain STs and/or ST complexes.

Pathogenic groups of ExPEC in relation to ST lineages are common worldwide. The most prevalent lineages of ExPEC are ST131/B2, ST127/B2, ST95/B2, ST73/B2, and ST69/D in the UK (Ciesielczuk et al., 2016). The most widespread lineages of ExPEC were ST95 followed by ST131 in the United States (Stephens et al., 2017). The B2/ST131 lineage is the most common in Italy (Cerquetti et al., 2010). Of the clonal groups of ST131, the percentage of Extended Spectrum β-lactamase (ESBL) producing ExPEC was 45.8% in Germany and 41% in southern of Europe and 39.9% in Turkey (Aktaş et al., 2017; Arvand, Moser & Pfeifer, 2013; Pomba et al., 2014).

ExPEC are generally encountered as urinary pathogens (Dale & Woodford, 2015). Accordingly, urinary tract-associated *E. coli* remain a health problem in Turkey (Koksal et al., 2017). Moreover, the prevalence of *E. coli* originating from urinary tract systems is over 50% in Turkey (Yilmaz et al., 2016a; Yilmaz et al., 2016b). Nevertheless, *E. coli* strains have been isolated from blood associated with bacteraemia in Turkey (Kaya et al., 2013).

A notable increase in antibiotic resistance generates difficulties in the treatment of extra intestinal infections caused by ExPEC (Cerquetti et al., 2010; Matsumura et al., 2017). ExPEC generally exhibits resistance to cephalosporins, fluoroquinolones, beta-lactams, and sulphonamides (Blanco et al., 2011; Feglo et al., 2013). In particular, CTX-M type ESBL producing ExPEC is significant in terms of public health. The ST131 lineages belong to the B2 group and are the most prevalent among ExPEC. Thus, ExPEC in the B2 phylogenetic group and ST131 lineages produce CTX-M type β-lactamase as well as fluoroquinolones resistant (Arvand, Moser & Pfeifer, 2013). Moreover, the H30 sub-lineage of ST131 has also emerged worldwide. The H30 sub-lineage of ST131 produces VIM-1 and KPC-3 carbapenemases in Italy (Accogli et al., 2014). Similarly, a high percentage of CTX-M type β-lactamase producing *E. coli* has been reported from Turkey (Aktaş et al., 2017).

ExPEC has a broad range of virulence factors (VFs), and they include genetically diverse strains. The VFs are encoded by chromosomal genes, and they are often located in pathogenicity islands (PAIs) or plasmids (Dale & Woodford, 2015). VFs are attributed to various bloodstream infections including urinary tract infection and septicaemia. The ExPEC is the main reason for infections out of the intestinal system. Thus, virulence factors generally comprise an iron acquisition system, surface structures, toxins and outer membrane proteins e.g., haemolysin, cytotoxic necrotizing factor, yersiniabactin, fimbriae, serum survival, and capsule synthesis (Koga et al., 2014; Subashchandrabose & Mobley, 2015).

Some ExPEC phylogenetic groups are responsible for various extraintestinal infections, and many ExPEC lineages are resistant to many antibiotics. The ExPECs studied here were isolated from blood samples in Turkish Hospital and had relationships among clonal lineages, antibiotic resistance, and virulence factors. Therefore, the aim of this study was to investigate phylogenetic relationship between virulence, clonal properties, and antibiotic resistance profiles among ExPEC isolates originated from Izmir Province in Turkey.

## Methods

### Bacterial isolates

**Figure 1:**
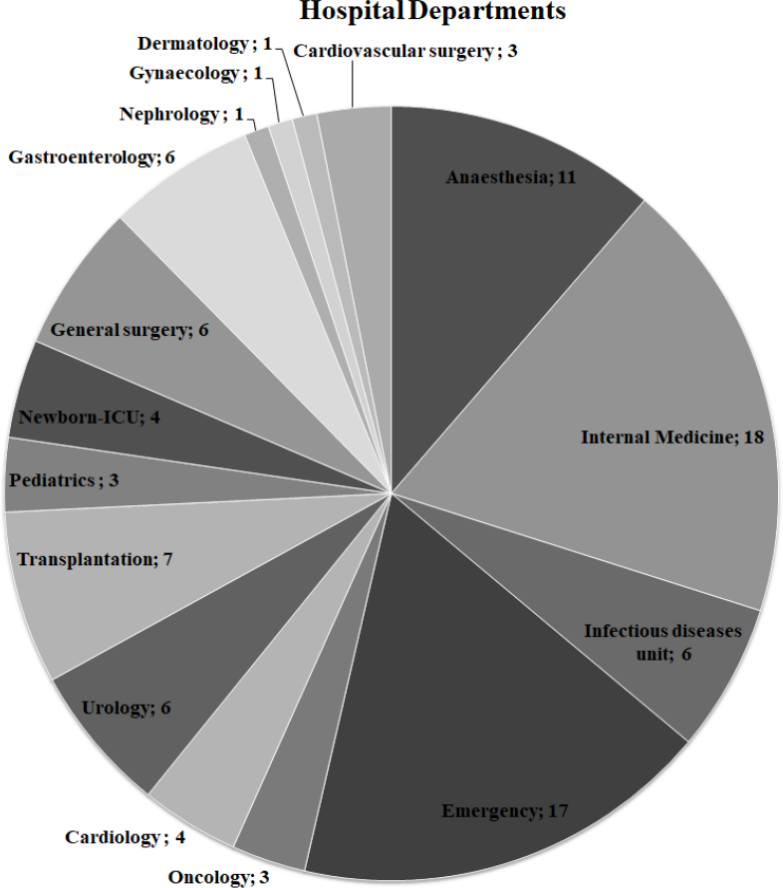
Distribution of hospital departments among ExPEC isolates. The number of departments where ExPEC isolates (*n* = 97) originated was shown on the pie chart. **Newborn-ICU**, Newborn- Intensive Care Unit

Presumed ExPEC isolates (*n* = 104) isolated from blood cultures were obtained from the bacterial culture collection of Ege University-Department of Medical Microbiology Laboratory. Ege University Hospital is a 1900–2000 bed capacity hospital. The clinical information on the ExPEC isolates is shown in Fig. 1. All isolates were identified by MALDI-TOF Mass Spectrometry (BioMérieux, Craponne, France). The isolates were stored in Luria Bertani Broth (LB) with 50% glycerol at −80 °C until further analysis. Colonies with a metallic green sheen were selected after culturing the strains on Eosin Methylene Blue Agar (EMB) plates (Merck, Kenilworth, NJ, USA). The control strains, *E. coli* CFTO73 and *E. coli* O25b:H4 (JJ1886), were kindly provided by Prof. James R. Johnson (University of Minnesota, VA Medical Centre, USA). *Escherichia coli* O25:H4 (DSM 22664 strain) was purchased from German Collection of Microorganisms and Cell Cultures. The quality control strain *E. coli* ATCC 25922 was obtained from Ege University-Department of Medical Microbiology.

### Antibiotic susceptibility testing

Antibiotic susceptibility and ESBL testing of the isolates was performed by an automated method (VITEK^®^2 BioMérieux). Susceptibility to 13 antibiotics including ampicillin, amoxicillin plus clavulanic acid, cefuroxime axetil, ceftriaxone, trimethoprim-sulfamethoxazole, cefepime, gentamicin, piperacillin/tazobactam, ciprofloxacin, amikacin, ertapenem, ceftazidime, and cefuroxime was tested using the Gram Negative Susceptibility card (AST-N325). Antibiotics for each isolate may vary because antibiotic susceptibility testing was applied to all ExPEC isolates within routine diagnosis. The McFarland standard was 0.5 (1.5 × 10^8^ cfu/ml). VITEK 2 cards were inoculated with *E. coli* bacterium via an integrated apparatus. The test tube with the bacterial suspension was placed into the cassette. Next, the AST card was inserted into the part of slot while inserting the transfer tube into the corresponding suspension tube. The prepared cassettes were transported into the chamber and incubated online at 35.5 + 1.0 °C. *Escherichia coli* ATCC 25922 was used as a control strain. Interpretation of the antibiotic susceptibility testing results was performed using the guidelines from the Clinical & Laboratory Standards Institute (CLSI, 2013). The susceptibility results were obtained and reported as resistant (R), sensitive (S) and intermediate resistant (I). The MIC breakpoint (µg/ml) of each antibiotic was as follow: ampicillin (R8,I:- S ≤ 8), amoxicillin plus clavulanic acid (R ≥ 8, *I* = 16 S ≤ 8), cefuroxime axetil (R ≥ 8, *I* = 16 S ≤ 8), ceftriaxone (R > 4, *I* = 2 and S ≤ 1), trimethoprim-sulfamethoxazole (R ≥4/76, I:- and S ≤2/38), cefepime (R ≥ 32, *I* = 16 and S ≤ 8), gentamicin (R ≥ 16, *I* = 8 and S ≤ 4), piperacillin/tazobactam (R ≥ 128, *I* = 32–64 and S ≤ 16), ciprofloxacin (R ≥ 4, *I* = 2 and S ≤ 1), amikacin (R ≥ 64, *I* = 32 and S ≤ 16), ertapenem (R ≥ 2, *I* = 1 and S ≤ 0.5), ceftazidime (R ≥ 16, *I* = 8 and S ≤ 4) and cefuroxime (R ≥ 32, *I* = 16, S ≤ 8).

### Genomic DNA Isolation

Isolation of the bacterial genomic DNA was performed using either the Wizard^®^Genomic DNA Purification Kit (Promega, Madison, WI, USA) or the JetFlex Genomic DNA Purification Kit (Thermo Fisher Scientific, Waltham, MA, USA). Plasmid DNA isolation was performed with a Pure Yield Plasmid MiniPrep System kit (Promega). Isolated DNA samples were stored at −20 °C for further analysis.

### 16S rRNA PCR

The 16S rRNA PCR analysis was modified from Frank et al. (2008). The 1,465 bp fragment of the 16S rRNA gene was amplified in a 50 µl reaction volume containing 1× PCR buffer (Thermo Fisher Scientific), 0.2 mM of each dNTP, 2 mM MgCl_2_, and 0.5 µM of each primer (16S rRNA universal primers: 27F and 1492R) (Table 1). The reaction conditions were of 2 min at 95 °C initial denaturation, 25 cycles of 1 min at 95 °C, 1 min at annealing temperature (55 °C), and 2 min at 72 °C followed by 10 min at 72 °C final extension. The PCR amplicons were analysed in agarose gel electrophoresis and visualized using the WiseDoc Gel Doc System WiseUV Transilluminator.

**Table 1:** Primers used in this study.

Gene	Sequence (5′ → 3′)	Size (bp)	Description	Reference
*chuA*	GAC GAA CCA ACG GTC AGG AT	279	Hemetransport in Enterohemorrhagic O157:H7 *E.coli*	Clermont, Bonacorsi & Bingen (2000)
	TGC CGC CAG TAC CAA AGA CA			
*yjaA*	TGA AGT GTC AGG AGA CGC TG	211	Protein of function unknown	Clermont, Bonacorsi & Bingen (2000)
	ATG GAG AAT GCG TTC CTC AAC			
*TSPE4.C2*	GAG TAA TGT CGG GGC ATT CA	152	Putative DNA fragment (TSPE4.C2) in *E. coli*	Clermont, Bonacorsi & Bingen (2000)
	CGC GCC AAC AAA GTA TTA CG			
*hlyA*	AAC AAG GAT AAG CAC TGT TCT GGC	1,777	Hemolysin	Koga et al. (2014)
	ACC ATA TAA GCG GTC ATT CCC GTC			
*cnf1*	AAGATGGAG TTT CCT ATGCAGGAG	498	Cytotoxic necrotizing factor 1	Johnson & Stell (2000)
	CAT TCA GAG TCC TGC CCT CAT TAT T			
*iutA*	GGC TGG ACA TCA TGG GAA CTG G	302	Aerobactin siderophore receptor	Koga et al. (2014)
	CGT CGG GAA CGG GTA GAA TCG			
*fyuA*	TGA TTA ACC CCG CGA CGG AA	880	Yersiniabactin	Johnson & Stell (2000)
	CGC AGT AGG CAC GAT CTT GTA			
*iroN*	AAT CCG GCA AAG AGA CGA ACC GCC T	553	Salmochelin siderophore receptor	Koga et al. (2014)
	GTT CGG GCA ACC CCT GCT TTG ACT TT			
*papG*	CTG TAA TTA CGG AAG TGA TTT CTG	1,070	P fimbriae	Johnson & Stell (2000)
	ACT ATC CGG CTC CGG ATA AAC CAT			
*sfaS*	GTG GAT ACG ACG ATT ACT GTG	240	Sfa fimbriae	Johnson & Stell (2000)
	CCG CCA GCA TTC CCT GTA TTC			
*iss*	CAG CAA CCC GAA CCA CTT GAT G	323	Episomal increased serum survival	Johnson et al. (2008)
	AGC ATT GCC AGA GCG GCA GAA			
*traT*	GGT GTG GTG CGA TGA GCA CAG	290	Serum resistance	Johnson & Stell (2000)
	CAC GGT TCA GCC ATC CCT GAG			
*ibeA*	AGG CAG GTG TGC GCC GCG TAC	170	Invasion of brain endothelium	Johnson & Stell (2000)
	TGG TGC TCC GGC AAA CCA TGC			
*kpsMT II*	GCGCAT TTGCTGATA CTGTTG	272	Capsule synthesis K1 e K5	Johnson & Stell (2000)
	CAT CCA GAC GAT AAG CAT GAG CA			
*ompT*	TCA TCC CGG AAG CCT CCC TCA CTA CTA T	496	Episomal outer membrane protease	Koga et al. (2014)
	TAG CGT TTG CTG CAC TGG CTT CTG ATA C			
*rfb*	TACCGACGACGCCGATCTG	300	O-typing O25b serotype	Clermont et al. (2008)
	TGCTATTCATTATGCGCAGC			
*PAI*	GGACATCCTGTTACAGCGCGCA	930	pathogenicity-associated island (PAI) marker	Johnson & Stell (2000)
	TCGCCACCAATCACAGCCGAAC			
*adk*	ATTCTGCTTGGCGCTCCGGG	583	adenylate kinase	Maluta et al. (2014), Wirth et al. (2006)
	CCGTCAACTTTCGCGTATTT			
*fumC*	TCACAGGTCGCCAGCGCTTC	806	fumarate hydratase	Maluta et al. (2014), Wirth et al. (2006)
	GTACGCAGCGAAAAAGATTC			
*gryB*	TCGGCGACACGGATGACGGC	911	DNA gyrase	Maluta et al. (2014), Wirth et al. (2006)
	ATCAGGCCTTCACGCGCATC			
*icd*	ATGGAAAGTAAAGTAGTTGTT CCGGCACA	878	isocitrate/isopropylmalate dehydrogenase	Maluta et al. (2014), Wirth et al. (2006)
	GGACGCAGCAGGATCTGTT			
*mdh*	ATGAAAGTCGCAGTCCTCGGCGCT GCTGGCGG	932	malate dehydrogenase	Maluta et al. (2014), Wirth et al. (2006)
	TTAACGAACTCCTGCCCCAGAGCG ATATCTTTCTT			
*purA*	CGCGCTGATGAAAGAGATGA	816	adenylosuccinate dehydrogenase	Maluta et al. (2014), Wirth et al. (2006)
	CATACGGTAAGCCACGCAGA			
*recA*	CGCATTCGCTTTACCCTGACC	780	ATP/GTP binding motif	Maluta et al. (2014), Wirth et al. (2006)
	AGCGTGAAGGTAAAACCTGTG			
*16S rRNA*	AGAGTTTGATCCTGGCTCAG	1,465	16S rRNA, partial gene	Suardana (2014)
	GGTTACCTTGTTACGACTT			

### DNA sequence analysis

The resulting partial 16S rRNA gene PCR products were purified by BMLabosis (Ankara, Turkey) using the ExoSap-IT (Affymetrix) kit. The purified samples were sent to Macrogen (Amesterdam, The Netherlands) where the fragments were unidirectionally sequenced using the ABI 3730XL automated sequencer (Applied Biosystems, Foster City, CA, USA), and the BigDye Terminator v3.1 Cycle Sequencing Kit (Applied Biosystems). The resulting sequence reads were aligned and trimmed using the SILVA (Quast et al., 2013) and DNA Baser Assembler Software programs, respectively. Before phylogenetic analysis, all ambiguously aligned regions and gaps were removed with trimAL (v1.4) using a heuristic best automated parameter “-automated1” and manually assessed (Capella-Gutierrez, Silla-Martinez & Gabaldon, 2009). The lengths of the good quality sequences ranged between 901 and 1,261 bp.

### Phylogenetic classification based on 16S rRNA sequences

Nucleotide BLAST analysis was performed followed by TrimAI analysis. All 16S rRNA gene sequences were deposited in the NCBI GenBank database (Dataset S1). To ensure a reliable identification, the sequence similarities were analysed using the web-based SINA alignment service with the following criteria: reject sequences below 90% identity to consensus *E. coli* sequence and sequences with <700 nt in length (Pruesse, Peplies & Glockner, 2012). The multiple sequence alignment of 16S rRNA partial sequences was performed via the MUSCLE (Edgar, 2004). Clustering method was Neighbor Joining implemented in MEGA 7.0. (Kumar, Stecher & Tamura, 2016). The phylogenetic tree was inferred by using Maximum Likelihood method based on the Hasegawa–Kishino–Yano model (Felsenstein, 1985). The bootstrap consensus tree was constructed from 1,000 replicates. The visualization and sub-tree analysis of ExPEC isolates were implemented in MEGA 7.0 (Kumar, Stecher & Tamura, 2016). Bootstrap cut-off value (≥75%) are given for each node. To compare the phylogenetic relationship of *E. coli* isolates with pathogenic and non-pathogenic ExPEC strains, The sub-tree analysis was involved 111 nucleotide sequences (ExPEC isolates (*n* = 97) and related reference ExPEC strains (*n* = 14)). The description and accession numbers of reference strains are shown in Table 2.

**Table 2:** Extra intestinal pathogenic *Escherichia coli* (ExPEC) reference strains.

ID_NCBI	Strain	Description	Reference
*Escherichia coli* AP009378	**SE15**	*Escherichia coli* strain SE11 (O152:H28) isolated from feces of a healthy adult and classified into *E. coli* phylogenetic group B1	Toh et al. (2010)
*Escherichia coli* HG941718	**EC958**	*Escherichia coli* ST131 Urinary tract infection	Forde et al. (2014)
*Escherichia coli* CP006784	**JJ1886**	Highly Virulent CTX-M-15-Producing H30-Rx Subclone of *Escherichia coli* ST131	Andersen et al. (2013)
*Escherichia coli* CP002797	**NA114**	Multidrug-Resistant Uropathogenic *Escherichia coli* Strain	Avasthi et al. (2011)
*Escherichia coli* AE014075	**CFT073**	Uropathogenic *Escherichia coli* strain	Welch et al. (2002)
*Escherichia coli* CU928162	**ED1a**	Commensal strain-isolated in the 2000s from the faeces of a healthy man in France	Touchon et al. (2009)
*Escherichia coli* CP000243	**UTI89**	UTI89 is a uropathogenic *Escherichia coli* (UPEC) belonging to phylogroup B2	Chen et al. (2006)
*Escherichia coli* CP000468	**APEC O1**	Avian pathogenic *Escherichia coli* strain isolated from chickens and turkeys clinically diagnosed with colibacillosis	Johnson et al. (2007)
*Escherichia coli* CU928161	**S88**	Isolated in 1999 from the cerebro-spinal fluid of a new born with late-onset neonatal meningitis in France	Touchon et al. (2009)
*Escherichia coli* CU928163.2	**UMN026**	Extraintestinal pathogenic *Escherichia coli* strain (ExPEC) isolated from an acute cystitis patient in the USA in 1999	Touchon et al. (2009)
*Escherichia coli* AP009240	**SE11**	A commensal bacterium was isolated from the feces of a healthy human adult. It belongs to the *E.coli* phylogenetic group B1	Oshima et al. (2008)
*Escherichia coli* CU928160	**IAI1**	Isolated from the faeces at 1980s in France	Touchon et al. (2009)
*Escherichia coli* CP000948	**DH10B**	Strain DH10B is a derivative of the already sequenced K12	Durfee et al. (2008)
*Escherichia coli* U00096	**K-12 (MG1655)**	*Escherichia coli* strain K-12 was obtained from a stool sample of a diphtheria patient	Bachmann (1996); Blattner et al. (1997)

### O25b typing

ExPEC isolates belonging to the Group B2 were evaluated for ST131 situation by O25b *rfb* detection (Clermont et al., 2009; Johnson et al., 2014). The allele-specific PCR, using the O25b type primers (Table 1) and the annealing temperature of 60 °C producing a PCR product of 300 bp was performed as described elsewhere (Clermont et al., 2008).

### Determination of the ExPEC phylogenetic groups

Phylogenetic classification of the ExPEC strains into the A, B1, B2 and D groups used triplex PCR, based on amplification of *chuA* (a gene required for heme transport in enterohemorrhagic O157:H7 *E. coli)*, *yjaA* (a gene detected from complete genome sequence of *E. coli* K-12 the function of which is unknown), and the DNA fragment TSPE4.C2 (from subtractive library of *E. coli*). The PCR used carried out in 50 µl final volume containing 1.25U DyNAzyme™ II DNA Polymerase (Thermo Fisher Scientific, Waltham, MA, USA) in 1×  PCR buffer, 0.2 µM of each dNTP, 2 mM MgCl_2_, and 1 µM of each primer (Table 1). The PCR program included denaturation at 95 °C for 4 min, followed by 30 cycles of 95 °C for 5 s and 55 °C for 10 s, with a final extension step at 72 °C for 5 min (Clermont, Bonacorsi & Bingen, 2000). During the determination of the phylogenetic classification of the ExPEC isolates, the *chuA* gene encoding heme transport in enterohemorragic O157:H7 was the first evaluation step for the assigning the B2 or D group.

### Virulence factors

A total of 13 virulence factors (VFs) encoded by chromosome or plasmid ,were investigated. The genes included the PAI marker, the adhesin genes (*papG*, *papC*, *sfaS*), the toxin genes (*hlyA*, *cnf1*), the capsule synthesis gene (*kpsII)*, the siderophore genes (*fyuA*, *iroN*, *iutA*), the serum resistance genes (*iss* and *traT*), the brain microvascular endothelium invasion gene (*ibeA*), and the outer membrane protein gene (*ompT*) (Table 1). The VF gene detection was performed in the triplex, duplex, and/or standard PCR analysis (Triplex PCR: **1.set:** *cnf1*, *papG* and *sfaS*, **2.set**: *fyuA*, *iutA* and *IroN*. Duplex PCR: **1. Set**: *hlyA* and *kpsII*, **2. Set:** *iss* and *traT*, 3. **Set:** *PAI* and *ibeA*. Standard PCR: *ompT*). PCR conditions were performed as follows: 1.25U DyNAzyme™II DNA Polymerase (Thermo Fisher Scientific) in 1x PCR buffer (Thermo Fisher Scientific), 0.2mM of each dNTP, 2 mM MgCl_2_, and 1 µM of each primer (Table 1). The PCR program consisted of a 5 min initial denaturation at 95 °C, followed by 30 cycles of 95 °C for 1min, annealing at 55–68 °C for 1min, and extension at 72 °C for 1 min, and a final extension step at 72 °C for 10 min (Koga et al., 2014). *E. coli* CFTO73, *E. coli* O25b:H4 (JJ1886), and *E. coli* DSM 22664 strains were used as a control strain during the amplification of virulence factors.

### Multi locus sequence typing

MLST analysis was used to the ST groups of the strains. PCR amplification and sequencing of seven housekeeping genes (*adk*, *fumC*, *gyrB*, *icd*, *mdh*, *purA* and *recA*) were carried according to the protocols obtained from (http://mlst.warwick.ac.uk/mlst/dbs/Ecoli). Primer sequences for MLST analysis were similarly obtained from http://mlst.warwick.ac.uk/mlst/dbs/ (Table 1). The reaction mixture (50 µl) included 1.25U Tag DNA Polymerase (Thermo Fisher Scientific) in 1× PCR buffer (Thermo Fisher Scientific), 0.2mM of each dNTP, 3 mM MgCl_2_, and 1 µM of each primer (Table 1). The PCR program consisted of a 2 min initial denaturation at 95 °C followed by 30 cycles of 1 min at 95 °C, 1 min at annealing temp (55–65 °C), and 2 min at 72 °C, followed by a 5 min final extension at 72 °C .The sequencing of the PCR amplicons was performed as described above. For the Sequence type assignment, amplicons were checked against to MLST sequence database (https://pubmlst.org/) (Jolley & Maiden, 2010). The accession numbers were deposited into the GenBank (MH279595 –MH279614 and MH561267 –MH561386).

## Results

Ninety-seven (93.26 %) of the obtained 104 tentative ExPEC isolates were confirmed to be *E. coli* based on 16S rRNA sequence analysis. The other isolates were identified as *Proteus mirabilis* (*n* = 2), *Shigella flexneri* (*n* = 2), and *Escherichia fergusonii* (*n* = 3). These the 97 ExPEC isolates were used for subsequent analysis.

The antibiotic susceptibility results (Table 3) showed that of the 97 strains 72 (74.22%) were ampicillin-resistant. A total of 31 isolates (31.95%) were resistant against the fluoroquinolone group antibiotic ciprofloxacin; 21 strains (21.65%) were susceptible to ampicillin and gentamicin (Dataset S2 and Table 3), and 53 strains (54.63%) produced ESBL. The antibiotic resistance of the ESBL-producing bacteria is shown in the Dataset S2.

**Table 3:** Antibiotic susceptibility profiles of the ExPEC isolates.

Antibiotic	Antibiotic susceptibility					
	R		I		S	
	*n*	%	*n*	%	*n*	%
Ampicillin	72	74.22	n.a.	n.a.	21	21.64
Amoxicillin clavulanic acid	38	39.17	n.a.	n.a.	n.a.	n.a.
Cefuroxime axetil	64	65.97	n.a.	n.a.	7	7.21
Ceftriaxone	56	57.73	n.a.	n.a.	n.a.	n.a.
Trimethoprim-sulfamethoxazole	55	56.70	n.a.	n.a.	20	41.7
Cefepime	47	48.45	6	6.18	n.a.	n.a.
Gentamicin	31	31.95	1	1.03	21	20.61
Piperacillin/tazobactam	29	29.89	16	16.49	n.a.	n.a.
Ciprofloxacin	31	31.95	1	1.03	8	8.24
Amikacin	n.a.	n.a.	18	18.55	2	2.06
Ertapenem	2	2.06	n.a.	n.a.	n.a.	n.a.
Ceftazidime	2	2.06	n.a.	n.a.	n.a.	n.a.
Cefuroxime	28	28.86	1	1.03	n.a.	n.a.

**Notes.**

n.a. not applicable R resistant I intermediate resistant S susceptible n number of ExPEC isolates

The phylogenetic classification of ExPEC isolates Clermont, Bonacorsi & Bingen (2000) is shown in Dataset S1. The most prevalent group was D with 37 strains (38.14%), followed by A, 29 strains (29.89%), B2 with 20 strains (20.61%), and B1 with 11 strains (11.34%). The *chuA* gene was observed in ExPEC isolates belonging to the B2 and D phylogenetic groups. Moreover, the *yjaA* gene was detected in only ExPEC isolates belonging to the B2 phylogenetic group. In other words, ExPEC isolates encoding *chuA* and *yjaA* genes were assigned to the B2 phylogenetic group (*n* = 20). The B2 phylogroup is associated with ST131 status (Johnson et al., 2010). Therefore, the B2 phylogroup isolates were examined for the presence of the O25b *rfb* allele and that was present in six strains (Dataset S1, highlighted in light grey). Importantly, four of the strains (ID: 6, 16, 28 and 103) were fluoroquinolone (ciprofloxacin) resistant that is characteristic for this emerging clonal group of ExPEC strains.

**Figure 2:**
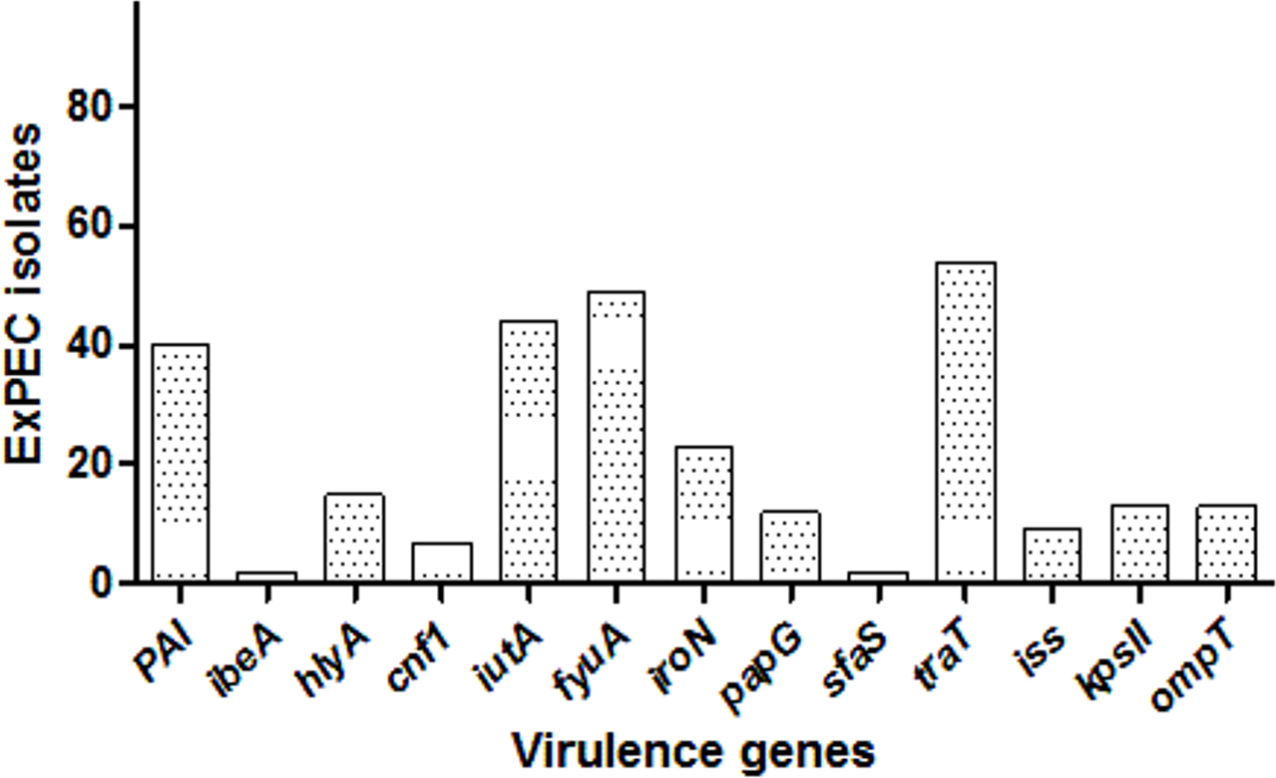
Distribution of virulence genes among ExPEC isolates. The bar plot was constructed for 97 ExPEC isolates to illustrate virulence genes distribution. The numbers presented at the top of bars demonstrated the number of virulence genes. *PAI:* pathogenicity-associated island (PAI) marker (*n* = 40), *ibeA*, Invasion of brain endothelium (*n* = 2); *hlyA*, hemolysin (*n* = 15); *cnf1*, cytotoxic necrotizing factor (*n* = 7); *iutA*, Aerobactin siderophore receptor (*n* = 44); *fyuA*, Yersiniabactin (*n* = 49); *iroN*, Samochelin siderophore receptor (*n* = 23); *papG*, P fimbriae (*n* = 12); *sfaS*, Sfa fimbriae (*n* = 2); iss, Episomal increased serum survival; *traT*, serum resistance (*n* = 54), (*n* = 9); *kpsII*, Capsule synthesis K1 and K5 (*n* = 13); *ompT*, Episomal outer membrane protease (*n* = 13).

Among the studied strains, the serum resistance gene *traT* was the most common (55.7%), followed by *fyuA* (50.5%), *iutA* (45.3%), PAI (41.2%), *iroN* (23.7.%), *hlyA* (15.4%), *kpsII* (13.4%), *ompT* (13.4%), *papG* (12.4%), *iss* (9.3%), *cnf1* (7.2%), *ibeA* (2.06%), and *sfaS* (2.06%). One isolate (ID:56) carried eight, three isolates had seven, and six isolates had six VF genes, and altogether 12 isolates carried none. The distribution of VF genes is shown in Dataset S1 and Fig. 2. The numbers of virulence factor-associated genes per each ExPEC isolate were varied.

The ExPEC isolates originated from various hospital departments (Fig. 1). The ExPEC isolates originating from internal medicine (*n* = 18) belonged to the phylogenetic groups B2, A, B1, and D. Of these, isolates including ID: 3, ID: 22, ID: 24 and ID: 99 belonged to the B2 phylogenetic group. The emergency department (*n* = 17) had the most ExPEC isolates followed by internal medicine. Similarly, isolates originating from the emergency department belonged to phylogenetic groups B2, A, B1, and D. Of these, isolates including ID: 5, ID: 19 and ID: 88 belonged to the B2 phylogenetic group.

**Figure 3:**
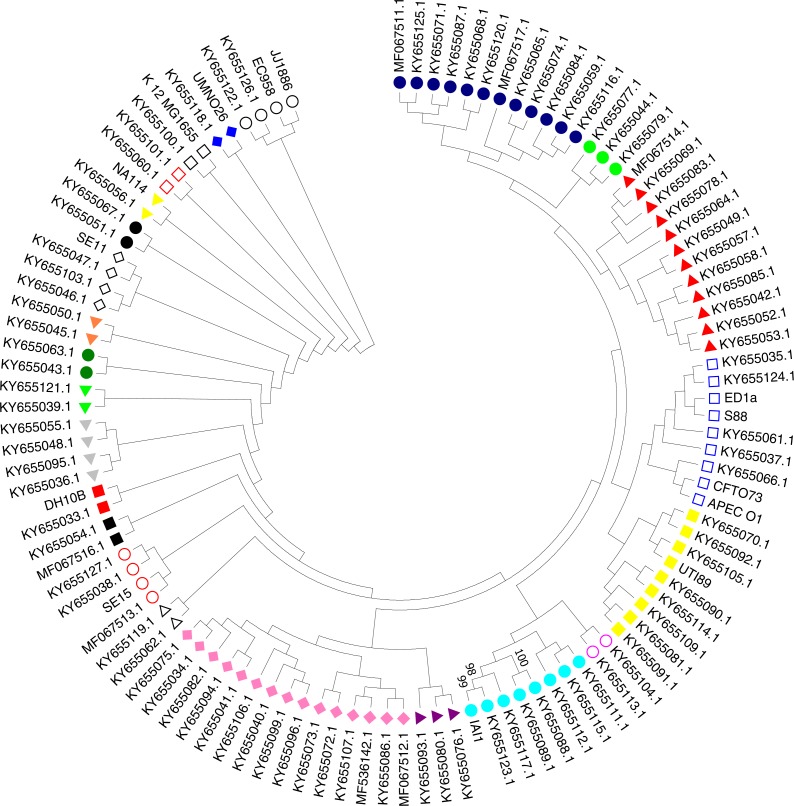
Phylogenetic tree of ExPEC blood culture isolates based on 16S rRNA analysis. The evolutionary history was inferred by using the Maximum Likelihood method based on the Hasegawa–Kishino–Yano model (Hasegawa, Kishino & Yano, 1985). The bootstrap consensus tree inferred from 1,000 replicates (Felsenstein, 1985) is taken to represent the evolutionary history of the taxa analyzed (Felsenstein, 1985). Branches corresponding to partitions reproduced in less than 50% bootstrap replicates are collapsed. Initial tree(s) for the heuristic search were obtained by applying the Neighbor-Joining method to a matrix of pairwise distances estimated using the Maximum Composite Likelihood (MCL) approach. The analysis involved 111 nucleotide sequences (97 ExPEC isolate sequences and of the 14 related ExPEC strain sequences fetched from the NCBI GenBank database). All positions containing gaps and missing data were eliminated. There were a total of 382 positions in the final dataset. Evolutionary analyses were conducted in MEGA7 (Kumar, Stecher & Tamura, 2016). The symbols shown in the tree indicative for the sub-clusters that were closely related (blue filled circle, green filled circle, and red filled triangle)-(empty blue square and filled yellow square)-(empty lilac circle and aqua filled circle)-(purple filled triangle and lilac filled rhomb)-(empty black triangle)-(empty red circle)-(black filled square)-(red filled square)-(grey filled triangle)-(lime filled triangle)-(dark green filled circle)-(orange filled triangle)-(empty black rhomb)-(black filled circle)-(yellow filled triangle)-(empty orange square)-(empty black square)-(blue filled rhomb)-(empty black circle).

A phylogenetic tree based on the maximum likelihood method under the Hasegawa-Kishino-Yano model of 16S rRNA sequences was generated for the 111 strains including the 97 studied ExPEC isolates and 14 ExPEC related strains (Fig. 3, Table 2). The main clusters that were similar were conserved. These were identified via internal branches (<0.0000).The phylogenetic tree revealed the presence of a large strain cluster among 97 ExPEC isolates. Under the 21 main clusters, there were 32 sub clusters. The ExPEC isolates shown in the phylogenetic tree with blue filled circle, green filled circles, and red filled triangles were closely related (Fig. 3). The sub-cluster with empty blue squares was clustered with some of reference ExPEC strains including ED1a, S88, CFTO73, and APECO1. These reference strains were a commensal strain isolated from a healthy human, a neonatal meningitis isolate, an uropathogenic *E. coli*, and avian pathogenic *E. coli*, respectively. The ExPEC isolates including ID:4 (B2, ESBL +), ID: 6 (B2, ESBL +), ID: 101 (B2, ESBL +), ID: 31 (A, ESBL-), and ID: 36 (B1, ESBL +) were clustered with these reference strains.

The sub-cluster shown with filled yellow squares included 8 ExPEC isolates together with UTI89 which is uropathogenic *E. coli* belonging to the phylogenetic group B2. These were clustered together, and these isolates were closely related to reference strain UTI89 (Fig. 3). However, they belonged to the different phylogenetic groups including D, A, and B1 within this sub-cluster. However, the sub-cluster shown with filled black squares was included two ExPEC isolates within the same phylogenetic tree (ID: 100 (B2, ESBL +) and ID: 24 (B2, ESBL +)). In the sub-group shown as empty circle, 2 ExPEC isolates (ID: 103 (B2, ESBL +) and ID: 98 (A, ESBL-)) were closely related to JJ1886 which is a ST131 clone (Fig. 3 and Table 2).

MLST analysis is a standard technique used to compare phylogroups and determine ExPEC lineages (Dale & Woodford, 2015). Thereby, MLST analysis of 20 ExPEC isolates (Isolate IDs: 3, 4, 5, 6, 8, 16, 17, 19, 22, 24, 28, 39, 53, 88, 95, 96, 99, 100, 101, and 103) belonging to the B2 phylogenetic group was carried out. The ST of 10 isolates out of 20 was identified as ST131. The others represented STs ST95, ST14, ST10, ST69, ST1722, ST141, ST88, ST80 and ST998 (https://pubmlst.org/). The ExPEC isolates identified as ST131 and related alleles were found to be ESBL producers (Table 4). However, some ExPEC isolates included in the B2 phylogenetic group (Isolate ID: 5 (ST95), 8 (ST80), 17 (ST14 and 96 (ST1722) had no antibiotic resistance. Insertion and/or deletion were detected in isolate ID:5 for the *adk* gene for allele 37. Other insertions and deletions were as follows: isolate ID:8 *adk* gene for allele 13, ID: 24 for the *adk* gene allele 53, ID:39 for the *adk* gene allele 53, ID:8 for the *recA* gene allele 10, and ID:16 for the *fumC* gene allele 40: 101^C^ →101^T^.

**Table 4:** Sequence Type classification and allelic profiles of the phylogroup B2 of the ExPEC isolates.

Isolate ID	ST	*adk*	*fumC*	*gyrB*	*icd*	*mdh*	*purA*	*recA*	clonal complex	Phylogenetic classification	ESBL (+/-)	O25
3	131	53	40	47	13	36	28	29	ST131 Cplx	B2	+	+
4	131	53	40	47	13	36	28	29	ST131 Cplx	B2	+	+
5	95	37	38	19	37	17	11	26	ST95 Cplx	B2	−	
6	131	53	40	47	13	36	28	29	ST131 Cplx	B2	+	+
8	80	13	24	19	14	23	1	10	ST568 Cplx	B2	−	
16	131	53	40	47	13	36	28	29	ST131 Cplx	B2	+	+
17	14	14	14	10	14	17	7	10	ST14 Cplx	B2	−	
19	10	550	11	4	8	8	8	2	ST10 Cplx	B2	+	
22	69	21	35	27	6	5	5	4	ST69 Cplx	B2	−	
24	131	53	40	47	13	36	472	29	ST131 Cplx	B2	+	
28	131	53	40	47	13	36	28	29	ST131 Cplx	B2	+	+
39	131	53	40	47	13	36	28	29	ST131 Cplx	B2	+	
53	1722	101	4	97	29	70	158	2	None	B2	−	
88	141	13	52	10	14	17	25	17	None	B2	−	
95	131	53	40	47	13	36	28	29	ST131 Cplx	B2	+	
96	1722	101	4	97	29	70	158	2	None	B2	−	
99	88	518	4	12	1	20	12	7	ST23 Cplx	B2	+	
100	131	53	40	25	13	36	28	29	ST131 Cplx	B2	+	
101	998	13	52	156	14	17	25	17	None	B2	+	
103	131	53	40	47	13	36	28	29	ST131 Cplx	B2	+	+

**Notes.**

ESBL Extended-Spectrum β-lactamase O25 O25 serotype of ExPEC ST sequence type

## Discussion

ExPEC is a very important bacterium causing many extraintestinal infections including urinary tract infections and fatal bacteraemia. The pathogenicity of these bacteria may depend on a variety of virulence determinants (Dale & Woodford, 2015). ExPEC represents many complex phylogenetic groups, and several clonal groups and lineages are associated with many different human extraintestinal infections (Fratamico et al., 2016). The presence of multiple antibiotic resistances in different phylogenetic groups demonstrates the importance of ExPEC in terms of public health. In this study, ExPEC isolates were characterized in terms of phylogenetic relationships, virulence determinants, sequence types, and antibiotic resistance. We detected that 74.22% of the ExPEC isolates were resistant to ampicillin, and over 50% of ExPEC isolates have produced ESBL. The most prevalent phylogroups was D and A. Serum resistance, yersiniabactin, and pathogenicity islands were the most commonly encountered virulence determinants. The ExPEC isolates assigned to ST131 lineage were shown to be ESBL producers. Isolates (ID: 6, 16, 28, and 103) belonging to the ST131 O25 clonal group were resistant to ciprofloxacin which is a fluoroquinolone. The ExPEC isolates were mostly originated from emergency and internal medicine departments.

Antibiotic resistance against sulphonamides, fluoroquinolones, aminoglycosides, β-lactams including cephalosporins, and carbapenems, has been encountered among the ExPEC isolates (Blanco et al., 2011; Cerquetti et al., 2010; Feglo et al., 2013; Ranjan et al., 2016). In addition to the ampicillin resistance (74.22%), isolates studied here showed resistance to cefepime (4th generation cephalosporin, 48.45%), gentamicin (aminoglycoside, 31.95%), and piperacillin-tazobactam (β-lactam and β-Lactamase inhibitor, 29.89%), and trimethoprim-sulfamethoxazole (sulphonamide 56.70%) (Table 3). Due to the heterogeneity of the ExPEC isolates seen in different studies, the observed antibiotic resistance distribution seen here could be differentiated among the ExPEC isolates. For example, the ciprofloxacin resistance rate was low in the Japanese study (Matsumura et al., 2017); here, it was 31.95% among the ExPEC isolates (Table 3).

The ESBL-producing ExPEC causes difficulties in the treatment of extraintestinal infections, and this has been noted all around the world (Arvand, Moser & Pfeifer, 2013; Padmini et al., 2017). The ESBL type CTX-M poses the most threat to the public health. The incidence rates are variable: In Ghana, 49.4% of nosocomial ExPEC produced ESBL (Feglo et al., 2013) but 54.63% in this study. In Turkey, the CTX-M type β-lactamase producing *E. coli* increased from 53% in 2008 (Yumuk et al., 2008) to 83.5% in 2017 (Aktaş et al., 2017). This is likely due to the horizontal gene transfer among *E. coli* (Park et al., 2012). In particular, *E. coli* ST131 has been drawn particular attention in accordance with higher virulence and resistance profiles including CTX-M-15 type ESBL producing a clonal lineage (Chung et al., 2012; Peirano, Costello & Pitout, 2010). The ST131, ST95, ST998, ST73, and ST69 have been most common clonal lineages worldwide (Alghoribi et al., 2015; Colpan et al., 2013; Doumith et al., 2015). Here, we detected 10 different ST of which ST131 was most common. The others are listed in Table 4. We note that, we did not encounter any resistant isolates among the ST14, ST95, ST80 and ST1722 clonal lineages (Dataset S2), in line with the observations that different ExPEC clonal lineages display different antibiotic susceptibilities (Doumith et al., 2015). Therefore, this could indicate that ExPEC in the same ST might be genetically diverse (Dale & Woodford, 2015).

Phylogenetic classification of ExPEC isolates can be performed based on the detection of the *chuA*, and *yjaA* genes, and particular DNA fragment known as TSPE4.C2 (Clermont, Bonacorsi & Bingen, 2000). Phylogenetic groups D and B2 of ExPEC have higher virulence in humans (Fratamico et al., 2016). Comparably, we detected D (38.14%, 37/97) and B2 (20.61%, 20/97) phylogenetic groups among ExPEC isolates. The detection rate of phylogenetic group A (29.8%, 29/97) was higher than phylogenetic group B2. In other studies, B2 was reported to be most common phylogenetic group in ExPEC (Koga et al., 2014; Micenkova et al., 2016). The B2 group has usually been associated with ST131 (Johnson et al., 2010). Our B2 phylogroup ExPECs were examined for the O25b *rfb* allele because serotype O25 and fluoroquinolone-resistant ExPECs are an emerging clonal group. This was present in 6 isolates (Dataset S1 light grey highlighted and Table 4). Of these, four (ID: 6, 16, 28 and 103) were fluoroquinolone resistant. Therefore, they may represent the ST131 O25 emerging clonal group (Dataset S2).

Virulence factors related to ExPEC pathogenicity are diverse and are generally divided into 5 main classes: adhesins, toxins, invasins, iron acquisition systems and capsule production. Finding a high prevalence of serum resistance factors among the strains is feasible because the strains were isolated from blood cultures where only serum-resistant pathogens could survive (Miajlovic et al., 2016). Iron uptake systems include aerobactin and yersiniabactin siderophores. These are necessary for the bacteraemia and the emergence of a successful infection (Dale & Woodford, 2015; Subashchandrabose & Mobley, 2015). A high incidence of aerobactin siderophore (*iutA*) and yersiniabactin (*fyuA*) genes was found in this study (Dataset S1 and Fig. 2). However, we observed no correlation between phylogenetic groups and virulence genes distribution. While the ExPEC strains belonging to the phylogenetic group A are generally considered to be commensal strains and non-pathogenic, virulence factor genes including *fyuA* were observed in phylogenetic group A in this research. Accordingly, Cyoia et al. (2015) reported that *fyuA* was detected in commensal strains of ExPEC, and PAI was detected in B2 compared to other phylogenetic groups of ExPEC (Cyoia et al., 2015). Similarly, a higher incidence of PAI was detected in ExPEC isolates belonging to the phylogenetic groups B2 and D than in B1 and A. Therefore, ExPEC virulence factors could be detected in commensal *E. coli* strains. It can be inferred from that the acquisition of virulence factors in ExPECs belonging to the phylogenetic group A is associated with horizontal gene transfer of the mobile genetic elements e.g., pathogenicity islands, plasmids and phages. For instance, pathogenicity islands mediates integration regions for the virulence genes thus acquisition of the virulence genes can be occurred in the genomic region (Leimbach, Hacker & Dobrindt, 2013).

Johnson & Stell (2000) stated that there were associations among VFs. For example, by the *pap* and *hlyA*, *fyuA* and *PAI* markers. The *hlyA* and *pap* are associated virulence factors among B2 strains but not all of the B2 strains (Johnson & Stell, 2000). A few ExPEC isolates belonging to the B2 group had *hlyA* along with the *pap* gene (ID: 8 and ID: 17). However, other B2 strains had only the *hlyA* gene (IDs: 3, 6, 8, 17, 18and 28). Our findings could demonstrate that not all B2 strains have both *hlyA* and *pap.* Both the *PAI* marker and *fyuA* were detected among ExPEC isolates belonging to the phylogenetic groups B2 and D (Dataset S1). Therefore, it can be concluded that we did not observe any clear a virulence association among the ExPEC isolates.

Virulence factors related to ExPEC pathogenicity are variable ranging from colonisation to invasion factors required for the establishment and prolonged infection. Virulence factors could be associated with infection site including urinary tract infection-associated VFs, neonatal meningitis-associated VFs and bacteraemia-associated VFs (Dale & Woodford, 2015). Moreover, the VFs of ExPECs could be a molecular marker for contribution to other pathotypes of *E. coli* strains. For example, enteroinvasive *E. coli* may include VFs and genetic PAI markers related to uropathogenic *E.coli* (Da Silva, De Mello Santos & Silva, 2017).

The 16S rRNA sequences in the 111 ExPEC strains (Fig. 3, Dataset S1 and Table 2) were used for phylogenetic analysis. The phylogenetic tree was obtained via the maximum likelihood method and demonstrated a large cluster among the 97 ExPEC isolates. The 16S rRNA sequences are highly conserved, and they could not discriminate between the ExPEC isolates, as noted also elsewhere (Lukjancenko, Wassenaar & Ussery, 2010). However, 16S rRNA is useful for the studying bacterial phylogeny and phylogenetic relationships (Suardana, 2014). Nevertheless, closely related ExPEC isolates were observed in a bootstrap consensus tree are shown with blue filled circles, green filled circles, and red filled triangles (Fig. 3). Although the phylogenetic groups were different, phylogenetic group B2, D, and B1 were clustered together. Similarly, a close relatedness was observed between reference strains and ExPEC isolates. For example, EC958 and JJ1886 were clustered together with B2 and A phylogenetic groups (ID: 103 and ID: 98). The related reference strains were shown with empty circles (Fig. 3). Therefore, we can deduce that the ExPEC isolates from different phylogenetic groups could be clustered together.

## Conclusions

The ExPEC strains has a broad range of virulence determinants and clonal lineages. Thus, certain problems can occur during the treatment of extraintestinal infections. An increased antibiotic resistance including β-lactamases could lead to the emerge of new pandemic clones. In line with other studies, we detected ampicillin resistance the most followed by cefuroxime axetil (cephalosporin) among ExPEC isolates included in this study. We also detected an antibiotic resistant ST131 clonal lineage along with other STs. These are among the most problematic clones worldwide. The detection of virulence factor genes in non-pathogenic phylogenetic groups A and B1 signified that the presence of the virulence genes might not be directly correlated to the phylogenetic group. Moreover, the high detection of serum resistance factors might be a prerequisite virulence factor for bacteria that cause systemic bloodstream infections. Also, yersinabactin could be a characteristic virulence factor for the bacteremic *E. coli* isolates. Moreover, ExPEC isolates belonging to the same phylogenetic group can have variable resistance and virulence properties.

##  Supplemental Information

10.7717/peerj.5470/supp-1 Dataset S1 16S rRNA gene sequence accession numbers, phylogenetic groups and virulence genes of the ExPEC isolates in this study **PG**: Phylogenetic Group

10.7717/peerj.5470/supp-2 Dataset S2 Antibiotic susceptibility testing profiles of the ExPEC isolates **ESBL** +: Extended-Spectrum *β*-Lactamase producing ExPEC isolates **Light Grey Highlighted Rows**: No Antibiotic resistance detected **S**, Susceptible; **I**, Intermediate Resistant In the non-highlighted rows, each ExPEC isolate was resistant to those antibiotics listed without the associated S and/or I label.

## Additional Information and Declarations

### DNA Deposition

The following information was supplied regarding the deposition of DNA sequences:
MF067517.1–MF067511.1; KY655127.1–KY655032.1; MH279595–MH279614; MH561267–MH561386.

### Data Availability

The following information was supplied regarding data availability: The raw data are provided in  Supplemental Datasets.
