# Supplementary material for: The relationship between phylogenetic classification, virulence and antibiotic resistance of extraintestinal pathogenic Escherichia coli in İzmir province, Turkey
Source: PeerJ. 2018 Aug 24;6:e5470. doi: 10.7717/peerj.5470 (PMC6110251; doi:10.7717/peerj.5470)
Supplement: Dataset S1 — PG: Phylogenetic Group [file peerj-06-5470-s001.docx]

| **Isolate ID** | **16S rRNA sequence Acc. No**. | **PG** | **Virulence genes** | | | | | | | | | | | | | |  |
| --- | --- | --- | --- | --- | --- | --- | --- | --- | --- | --- | --- | --- | --- | --- | --- | --- | --- |
|  |  |  | ***n* pos** | **PAI** | ***ibeA*** | ***hlyA*** | ***cnf-1*** | ***iutA*** | ***fyuA*** | ***iroN*** | ***papG*** | ***sfaS*** | ***traT*** | ***Iss*** | ***KpsII*** | ***OmpT*** | ***rfb.1bis*** |
| **2** | KY655033 | **D** | 4 | **+** |  |  |  |  | **+** |  |  |  | **+** |  | **+** |  |  |
| **3** | KY655034 | **B2** | 5 | **+** |  | **+** | **+** | **+** |  |  |  |  | **+** |  |  |  | **O25b** |
| **4** | KY655035 | **B2** | 3 | **+** |  |  |  | **+** |  |  |  |  | **+** |  |  |  | **O25b** |
| **5** | KY655036 | **B2** | 7 | **+** |  |  |  | **+** | **+** | **+** |  |  | **+** |  | **+** | **+** |  |
| **6** | KY655037 | **B2** | 6 | **+** |  | **+** | **+** | **+** | **+** |  |  |  | **+** |  |  |  | **O25b** |
| **8** | KY655038 | **B2** | 6 | **+** | **+** | **+** | **+** | **+** |  |  |  |  |  |  | **+** |  |  |
| **9** | KY655039 | **A** | 4 |  |  |  |  | **+** |  | **+** |  |  | **+** | **+** |  |  |  |
| **10** | KY655040 | **A** | 3 |  |  |  |  | **+** | **+** |  |  |  | **+** |  |  |  |  |
| **11** | KY655041 | **A** | 1 |  |  |  |  |  |  |  |  |  | **+** |  |  |  |  |
| **12** | KY655042 | **B1** | 0 |  |  |  |  |  |  |  |  |  |  |  |  |  |  |
| **13** | KY655043 | **D** | 5 |  |  |  |  | **+** | **+** | **+** |  |  | **+** | **+** |  |  |  |
| **14** | KY655044 | **D** | 1 |  |  |  |  |  | **+** |  |  |  |  |  |  |  |  |
| **15** | KY655045 | **A** | 2 | **+** |  |  |  |  |  |  |  |  | **+** |  |  |  |  |
| **16** | KY655046 | **B2** | 5 | **+** |  |  |  | **+** | **+** |  |  |  | **+** |  | **+** |  | **O25b** |
| **17** | KY655047 | **B2** | 6 | **+** |  | **+** |  | **+** | **+** |  |  |  | **+** |  | **+** |  |  |
| **18** | KY655048 | **A** | 5 |  |  | **+** |  | **+** | **+** | **+** |  |  | **+** |  |  |  |  |
| **19** | KY655049 | **B2** | 2 |  |  |  |  | **+** | **+** |  |  |  |  |  |  |  |  |
| **20** | KY655050 | **B1** | 1 |  |  |  |  |  |  |  |  |  | **+** |  |  |  |  |
| **21** | KY655051 | **A** | 6 | **+** |  | **+** | **+** | **+** | **+** |  |  |  | **+** |  |  |  |  |
| **22** | KY655052 | **B2** | 2 |  |  |  |  |  |  |  |  |  | **+** |  |  | **+** |  |
| **23** | KY655053 | **D** | 2 | **+** |  |  |  |  | **+** |  |  |  |  |  |  |  |  |
| **24** | KY655054 | **B2** | 4 | **+** |  |  |  | **+** | **+** |  |  |  | **+** |  |  |  |  |
| **25** | KY655055 | **D** | 2 |  |  |  |  | **+** | **+** |  |  |  |  |  |  |  |  |
| **26** | KY655056 | **D** | 2 |  |  |  |  |  | **+** |  |  |  |  |  | **+** |  |  |
| **27** | KY655057 | **D** | 2 |  |  |  |  | **+** | **+** |  |  |  |  |  |  |  |  |
| **28** | KY655058 | **B2** | 4 | **+** |  | **+** |  |  | **+** |  |  |  | **+** |  |  |  | **O25b** |
| **29** | KY655059 | **B1** | 0 |  |  |  |  |  |  |  |  |  |  |  |  |  |  |
| **30** | KY655060 | **B1** | 2 |  |  |  |  | **+** | **+** |  |  |  |  |  |  |  |  |
| **31** | KY655061 | **A** | 4 |  |  |  |  |  | **+** |  |  |  | **+** | **+** |  | **+** |  |
| **32** | KY655062 | **A** | 3 |  |  |  |  | **+** | **+** | **+** |  |  |  |  |  |  |  |
| **33** | KY655063 | **D** | 2 |  |  |  |  |  | **+** |  |  |  | **+** |  |  |  |  |
| **34** | KY655064 | **A** | 1 |  |  |  |  |  | **+** |  |  |  |  |  |  |  |  |
| **35** | KY655065 | **A** | 0 |  |  |  |  |  |  |  |  |  |  |  |  |  |  |
| **36** | KY655066 | **B1** | 4 |  |  |  |  | **+** |  | **+** |  |  | **+** |  |  | **+** |  |
| **37** | KY655067 | **B1** | 3 |  |  | **+** |  |  | **+** | **+** |  |  |  |  | **+** |  |  |
| **38** | KY655068 | **A** | 4 |  |  | **+** |  |  | **+** | **+** |  |  | **+** |  |  |  |  |
| **39** | KY655069 | **B2** | 4 | **+** |  |  |  | **+** | **+** |  |  |  | **+** |  |  |  |  |
| **40-a** | KY655070 | **D** | 3 |  |  |  |  | **+** | **+** |  |  |  | **+** |  |  |  |  |
| **40** | KY655071 | **D** | 3 | **+** |  | **+** |  |  |  |  |  |  |  |  | **+** |  |  |
| **41** | KY655072 | **D** | 1 | **+** |  |  |  |  |  |  |  |  |  |  |  |  |  |
| **42** | MF067511.1 | **A** | 1 |  |  |  |  |  | **+** |  |  |  |  |  |  |  |  |
| **44** | KY655073 | **A** | 1 |  |  |  |  |  | **+** |  |  |  |  |  |  |  |  |
| **45** | KY655074 | **A** | 2 |  |  |  |  |  | **+** |  |  |  | **+** |  |  |  |  |
| **46** | KY655075 | **A** | 3 | **+** |  |  |  | **+** |  |  |  |  | **+** |  |  |  |  |
| **47** | KY655076 | **D** | 2 |  |  | **+** |  |  |  |  |  |  |  |  | **+** |  |  |
| **48** | KY655077 | **A** | 3 | **+** |  |  |  |  |  |  |  |  | **+** |  | **+** |  |  |
| **49** | KY655078 | **D** | 1 | **+** |  |  |  |  |  |  |  |  |  |  |  |  |  |
| **50** | KY655079 | **D** | 1 | **+** |  |  |  |  |  |  |  |  |  |  |  |  |  |
| **51** | KY655080 | **D** | 3 | **+** |  | **+** |  |  |  |  |  |  |  |  | **+** |  |  |
| **52** | KY655081 | **D** | 7 | **+** |  |  |  | **+** | **+** | **+** | **+** |  | **+** |  |  | **+** |  |
| **53** | KY655082 | **B2** | 4 | **+** |  |  |  | **+** |  |  |  |  | **+** |  |  | **+** |  |
| **54** | KY655083 | **D** | 0 |  |  |  |  |  |  |  |  |  |  |  |  |  |  |
| **55** | KY655084 | **A** | 3 | **+** |  |  |  |  | **+** |  |  |  | **+** |  |  |  |  |
| **56** | KY655085 | **D** | 8 |  |  | **+** |  | **+** | **+** | **+** | **+** |  | **+** |  | **+** | **+** |  |
| **57** | KY655086 | **D** | 5 |  |  |  |  | **+** |  | **+** | **+** |  | **+** |  |  | **+** |  |
| **58** | KY655087 | **D** | 4 |  |  |  |  | **+** | **+** |  | **+** |  | **+** |  |  |  |  |
| **59** | KY655088 | **B1** | 2 | **+** |  |  |  |  |  |  | **+** |  |  |  |  |  |  |
| **60** | KY655089 | **D** | 3 |  |  | **+** |  | **+** |  |  |  |  | **+** |  |  |  |  |
| **61** | KY655090 | **D** | 5 |  |  |  | **+** | **+** | **+** |  | **+** |  | **+** |  |  |  |  |
| **62** | KY655091 | **D** | 3 |  |  |  |  |  | **+** |  |  |  | **+** |  | **+** |  |  |
| **63** | KY655092 | **D** | 2 |  |  |  |  |  | **+** |  |  |  | **+** |  |  |  |  |
| **64** | KY655093 | **D** | 4 | **+** |  |  |  | **+** | **+** |  |  |  | **+** |  |  |  |  |
| **65** | KY655094 | **D** | 0 |  |  |  |  |  |  |  |  |  |  |  |  |  |  |
| **66** | KY655095 | **A** | 0 |  |  |  |  |  |  |  |  |  |  |  |  |  |  |
| **67** | KY655096 | **A** | 0 |  |  |  |  |  |  |  |  |  |  |  |  |  |  |
| **70** | KY655099 | **A** | 2 |  |  |  |  | **+** |  |  |  |  | **+** |  |  |  |  |
| **71** | KY655100 | **D** | 0 |  |  |  |  |  |  |  |  |  |  |  |  |  |  |
| **72** | MF067512.1 | **A** | 2 |  |  |  |  |  | **+** |  |  |  | **+** |  |  |  |  |
| **73** | MF067513.1 | **D** | 4 |  |  |  |  |  | **+** | **+** | **+** |  | **+** |  |  |  |  |
| **74** | KY655101 | **A** | 3 |  |  |  |  | **+** | **+** |  |  |  | **+** |  |  |  |  |
| **75** | MF536142 | **A** | 2 |  |  |  |  |  |  |  |  |  | **+** | **+** |  |  |  |
| **76** | MF067514.1 | **A** | 7 | **+** |  |  |  | **+** | **+** | **+** | **+** |  | **+** |  |  | **+** |  |
| **79** | KY655103 | **D** | 0 |  |  |  |  |  |  |  |  |  |  |  |  |  |  |
| **80** | KY655104 | **B1** | 4 |  |  |  |  | **+** |  | **+** |  |  |  | **+** |  | **+** |  |
| **81** | KY655105 | **A** | 4 | **+** |  |  |  |  | **+** |  | **+** |  | **+** |  |  |  |  |
| **82** | KY655106 | **D** | 4 |  |  |  |  | **+** | **+** | **+** |  |  |  | **+** |  |  |  |
| **83** | KY655107 | **B1** | 3 |  |  |  |  | **+** |  | **+** |  |  |  | **+** |  | **+** |  |
| **85** | KY655109 | **B1** | 5 |  |  |  |  | **+** | **+** | **+** |  |  |  | **+** |  | **+** |  |
| **87** | KY655111 | **D** | 3 | **+** |  |  |  | **+** |  |  |  |  | **+** |  |  |  |  |
| **88** | KY655112 | **B2** | 6 | **+** |  |  | **+** |  | **+** | **+** | **+** | **+** |  |  |  |  |  |
| **89** | KY655113 | **A** | 2 |  |  |  |  | **+** |  |  |  |  | **+** |  |  |  |  |
| **90** | KY655114 | **A** | 0 |  |  |  |  |  |  |  |  |  |  |  |  |  |  |
| **91** | KY655115 | **D** | 1 | **+** |  |  |  |  |  |  |  |  |  |  |  |  |  |
| **92** | KY655116 | **D** | 0 |  |  |  |  |  |  |  |  |  |  |  |  |  |  |
| **93** | KY655117 | **B1** | 2 | **+** |  |  |  |  |  |  |  |  | **+** |  |  |  |  |
| **94** | KY655118 | **D** | 2 |  |  |  |  | **+** |  |  |  |  | **+** |  |  |  |  |
| **95** | KY655119 | **B2** | 4 | **+** |  |  |  | **+** |  | **+** |  |  | **+** |  |  |  |  |
| **96** | KY655120 | **B2** | 1 | **+** |  |  |  |  |  |  |  |  |  |  |  |  |  |
| **97** | KY655121 | **A** | 3 | **+** |  |  |  | **+** |  |  |  |  | **+** |  |  |  |  |
| **98** | KY655122 | **A** | 0 |  |  |  |  |  |  |  |  |  |  |  |  |  |  |
| **99** | KY655123 | **B2** | 5 |  |  |  |  | **+** | **+** | **+** |  |  |  | **+** |  | **+** |  |
| **100** | MF067516.1 | **B2** | 3 |  |  |  |  |  | **+** | **+** | **+** |  |  |  |  |  |  |
| **101** | KY655124 | **B2** | 6 | **+** | **+** |  | **+** |  |  | **+** |  | **+** | **+** |  |  |  |  |
| **102** | KY655125 | **D** | 1 | **+** |  |  |  |  |  |  |  |  |  |  |  |  |  |
| **103** | KY655126 | **B2** | 4 | **+** |  |  |  | **+** |  |  | **+** |  | **+** |  |  |  | **O25b** |
| **104** | MF067517.1 | **D** | 3 | **+** |  |  |  |  | **+** |  |  |  | **+** |  |  |  |  |
| **105** | KY655127 | **D** | 4 | **+** |  | **+** |  |  | **+** | **+** |  |  |  |  |  |  |  |
