# Supplementary material for: The relationship between phylogenetic classification, virulence and antibiotic resistance of extraintestinal pathogenic Escherichia coli in İzmir province, Turkey
Source: PeerJ. 2018 Aug 24;6:e5470. doi: 10.7717/peerj.5470 (PMC6110251; doi:10.7717/peerj.5470)
Supplement: Dataset S2 — ESBL +: Extended-Spectrum β-Lactamase producing ExPEC isolates Light Grey Highlighted Rows: No Antibiotic resistance detected S, Susceptible; I, Intermediate Resistant In the non-highlighted rows, each ExPEC isolate was resistant to those antibiotics listed without the associated S and/or I label. [file peerj-06-5470-s002.docx]

| **Isolate ID** | **Antibiotic Susceptibility** |
| --- | --- |
| **2** | ESBL+, Ampicillin, Amoxicillin clavulanic acid, Piperacillin/Tazobactam, Cefuroxime axetil, Ceftriaxone,Cefepime, Gentamicin, Trimethoprim-sulfamethoxazole |
| **3** | ESBL+, Ampicillin,Amoxicillin clavulanic acid, Cefuroxime axetil, Ceftriaxone, Cefepime, Gentamicin, Trimethoprim-sulfamethoxazole |
| **4** | ESBL+, Ampicillin, Amoxicillin clavulanic acid, Cefuroxime axetil, Ceftriaxone,Trimethoprim-sulfamethoxazole |
| **5** | Ampicillin:S,Gentamicin:S,Trimethoprim-sulfamethoxazole:S |
| **6** | ESBL +, Amoxicillin clavulanic acid, Ampicillin, Gentamicin, Piperacillin/tazobactam, Cefepime, Ceftriaxone, Cefuroxime axetil, Ciprofloxacin,Trimethoprim-sulfamethoxazole |
| **8** | Ampicillin:S, Gentamicin:S, Trimethoprim-sulfamethoxazole:S,Cefuroxime, Cefuroxime axetil:S |
| **9** | ESBL+,Amoxicillin clavulanic acid, Ampicillin, Gentamicin, Piperacillin/tazobactam,Cefepime, Ceftriaxone, Cefuroxime axetil, Ciprofloxacin, Trimethoprim-sulfamethoxazole |
| **10** | ESBL+, Ampicillin,Amoxicillin clavulanic acid,Piperacillin/Tazobactam, Cefuroxime axetil, Ceftriaxone, Cefepime, Gentamicin,Trimethoprim-sulfamethoxazole |
| **11** | ESBL+,Amoxicillin clavulanic acid, Ampicillin, Gentamicin, Piperacillin/tazobactam,Cefepime, Ceftriaxone,Cefuroxime, Cefuroxime axetil, Ciprofloxacin, Trimethoprim-sulfamethoxazole |
| **12** | Ampicillin:S,Gentamicin:S,Trimethoprim-sulfamethoxazole:S |
| **13** | ESBL+, Amoxicillin clavulanic acid,Ampicillin, Ertapenem, Piperacillin/tazobactam, Ceftazidime,Cefuroxime, Cefuroxime axetil, Trimethoprim-sulfamethoxazole |
| **14** | ESBL+, Amoxicillin clavulanic acid, Ampicillin,Ertapenem, Gentamicin, Piperacillin/tazobactam, Ceftazidime, Ceftriaxone,Cefuroxime, Cefuroxime axetil, Trimethoprim-sulfamethoxazole |
| **15** | ESBL+, Ampicillin, Amoxicillin clavulanic acid, Cefuroxime, Cefuroxime axetil, Ceftriaxone,Trimethoprim-sulfamethoxazole |
| **16** | ESBL+, Ampicillin,Piperacillin/Tazobactam, Cefuroxime, Cefuroxime axetil, Ceftriaxone,Cefepime,Ciprofloxacin,Trimethoprim-sulfamethoxazole |
| **17** | Ampicillin:S, Gentamicin:S, Trimethoprim-sulfamethoxazole:S, Cefuroxime axetil:S |
| **18** | ESBL+, Ampicillin,Amoxicillin clavulanic acid, Cefuroxime axetil, Ceftriaxone,Cefepime, Gentamicin |
| **19** | ESBL+, Amoxicillin clavulanic acid, Ampicillin,Piperacillin/tazobactam,Cefepime, Ceftriaxone, Cefuroxime axetil |
| **20** | ESBL+, Amoxicillin clavulanic acid, Ampicillin, Piperacillin/tazobactam,Cefepime,Ceftriaxone,Cefuroxime axetil, Ciprofloxacin, Trimethoprim-sulfamethoxazole |
| **21** | ESBL+, Ampicillin,Amoxicillin clavulanic acid, Cefuroxime axetil, Ceftriaxone, Cefepime, Gentamicin, Trimethoprim-sulfamethoxazole |
| **22** | Trimethoprim-sulfamethoxazole, Ampisillin, Amoxicillin clavulanic acid |
| **23** | Amikacin (I) |
| **24** | ESBL+, Amoxicillin clavulanic acid, Ampicillin, Piperacillin/tazobactam,Cefepime,Ceftriaxone,Cefuroxime axetil, Ciprofloxacin, Trimethoprim-sulfamethoxazole |
| **25** | Ampicillin,Amoxicillin clavulanic acid,Piperacillin/tazobactam (I), Trimethoprim-sulfamethoxazole |
| **26** | Ampicillin:S, Gentamicin:S, Trimethoprim-sulfamethoxazole:S |
| **27** | Ampicillin, Amoxicillin clavulanic acid, Trimethoprim-sulfamethoxazole |
| **28** | ESBL+, Ampicillin, Amoxicillin clavulanic acid, Cefuroxime, Cefuroxime axetil, Ceftriaxone,Cefepime,Ciprofloxacin, Trimethoprim-sulfamethoxazole |
| **29** | Ampicillin:S, Gentamicin:S, Trimethoprim-sulfamethoxazole:S |
| **30** | Ampicillin:S,Gentamicin:S,Trimethoprim-sulfamethoxazole:S |
| **31** | Ampicillin, Amoxicillin clavulanic acid, Gentamicin, Ciprofloxacin |
| **32** | Amoxicillin clavulanic acid, Ampicillin, Gentamicin,Piperacillin/Tazobactam, seftirakson, Trimethoprim-sulfamethoxazole |
| **33** | Ampicillin,Amoxicillin clavulanic acid,Piperacillin/tazobactam (I), Trimethoprim-sulfamethoxazole |
| **34** | ESBL+, Ampicillin, Amoxicillin clavulanic acid,Piperacillin/tazobactam, Cefuroxime, Cefuroxime axetil, Ceftriaxone,Cefepime, Gentamicin,Trimethoprim-sulfamethoxazole |
| **35** | Ampicillin:S, Gentamicin:S, |
| **36** | ESBL+, Ampicillin, Amoxicillin clavulanic acid,Cefepime,Ceftriaxone, Cefuroxime axetil, Ciprofloxacin, Gentamicin, Trimethoprim-sulfamethoxazole |
| **37** | Ampicillin:S, Gentamicin:S, Trimethoprim-sulfamethoxazole:S, Ciprofloxacin:S |
| **38** | ESBL+, Ampicillin,Amoxicillin clavulanic acid, Cefuroxime axetil, Ceftriaxone,Cefepime, Gentamicin,Trimethoprim-sulfamethoxazole |
| **39** | ESBL+, Ampicillin, Amoxicillin clavulanic acid, Cefepime,Ceftriaxone, Cefuroxime axetil, Ciprofloxacin, Trimethoprim-sulfamethoxazole |
| **40-a** | ESBL+, Ampicillin, Amoxicillin clavulanic acid, Piperacillin/tazobactam,Cefuroxime axetil, Ceftriaxone,Cefepime, Gentamicin, Trimethoprim-sulfamethoxazole |
| **40** | ESBL+,Cefepime, Gentamicin, Ciprofloxacin, Trimethoprim-sulfamethoxazole, Ampicillin, Amoxicillin clavulanic acid, Piperacillin/Tazobactam(I), Cefuroxime axetil, Ceftriaxone |
| **41** | ESBL+, Ampicillin, Amikacin (I) Amoxicillin clavulanic acid, Piperacillin/tazobactam(I),Cefepime,Ceftriaxone, Cefuroxime axetil, Ciprofloxacin, Trimethoprim-sulfamethoxazole |
| **42** | Ampicillin:S, Gentamicin:S, Cefuroxime,Cefuroxime axetil :S, Trimethoprim-sulfamethoxazole:S, |
| **44** | Ampicillin, Piperacillin/Tazobactam, Cefuroxime,Cefuroxime axetil,Cefepime, Ciprofloxacin, Trimethoprim-sulfamethoxazole, Amoxicillin clavulanic acid |
| **45** | Ampicillin, Amoxicillin clavulanic acid |
| **46** | ESBL+, Ampicillin, Amikacin (I) Amoxicillin clavulanic acid, Piperacillin/tazobactam(I),Cefepime,Ceftriaxone, Cefuroxime axetil, Ciprofloxacin, Trimethoprim-sulfamethoxazole |
| **47** | ESBL+, Ampicillin,Cefuroxime,Cefuroxime axetil,Cefepime, Ceftriaxone, Ciprofloxacin (I), Trimethoprim-sulfamethoxazole, Amoxicillin clavulanic acid |
| **48** | Ampicillin:S, Gentamicin:S,Cefuroxime,Cefuroxime axetil :S, Ciprofloxacin:S, Trimethoprim-sulfamethoxazole:S, |
| **49** | ESBL+, Ampicillin,Cefuroxime,Cefuroxime axetil,Cefepime, Ceftriaxone, Trimethoprim-sulfamethoxazole |
| **50** | ESBL+,Cefepime, Ampicillin,Amoxicillin clavulanic acid, Cefuroxime axetil,Ceftriaxone, |
| **51** | Ampicillin:S, Gentamicin:S, Cefuroxime,Cefuroxime axetil :S, Ciprofloxacin:S, Trimethoprim-sulfamethoxazole:S, |
| **52** | Ampicillin,Amoxicillin clavulanic acid,Gentamicin |
| **53** | Ampicillin,Amoxicillin clavulanic acid, Piperacillin/tazobactam, Trimethoprim-sulfamethoxazole |
| **54** | ESBL+, Trimethoprim-sulfamethoxazole, Ampicillin, Amoxicillin clavulanic acid, Piperacillin/Tazobaktam (I), Cefuroxime axetil, Ceftriaxone,Cefepime |
| **55** | ESBL+, Ampicillin, Cefuroxime axetil, Ceftriaxone,Cefepime (I) |
| **56** | ESBL+, Ampicillin, Cefuroxime axetil, Ceftriaxone,Cefepime |
| **57** | Ampicillin:S, Gentamicin:S,Cefuroxime, Cefuroxime axetil:S, Trimethoprim-sulfamethoxazole:S, |
| **58** | Trimethoprim-sulfamethoxazole, Ciprofloxacin,Ampicillin, Piperacillin/Tazobaktam, Cefuroxime axetil, Ceftriaxone,Cefepime |
| **59** | Ampicillin |
| **60** | ESBL+, Amikacin (I), Gentamicin, Trimethoprim-sulfamethoxazole, Ampicillin, Cefuroxime axetil, Ceftriaxone,Cefepime, |
| **61** | ESBL+,Cefepime, Amikacin (I), Gentamicin, Ciprofloxacin, Piperacillin/Tazobaktam(I), Cefuroxime axetil,Ceftriaxone, Ampicillin |
| **62** | ESBL+,Cefepime, Amikacin (I), gentamsin, Ciprofloxacin, Piperacillin/Tazobaktam, Cefuroxime axetil,Ceftriaxone, Ampicillin,Trimethoprim-sulfamethoxazole |
| **63** | ESBL+, Gentamicin, Trimethoprim-sulfamethoxazole,Cefepime, Ampicillin, Piperacillin/Tazobactam (I), Cefuroxime axetil, Ceftriaxone |
| **64** | ESBL+,Cefepime, Amikacin (I), Gentamicin, Ciprofloxacin, Trimethoprim-sulfamethoxazole, Ampicillin, Piperacillin/Tazobactam, Cefuroxime axcetil, Ceftriaxone |
| **65** | Ampicillin:S, Gentamicin:S,Cefuroxime, Cefuroxime axetil :S, Ciprofloxacin:S, Trimethoprim-sulfamethoxazole:S, |
| **66** | Ampicillin:S, Gentamicin:S, Cefuroxime,Cefuroxime axetil :S, Trimethoprim-sulfamethoxazole:S, |
| **67** | Ampicillin, Gentamicin, Trimethoprim-sulfamethoxazole |
| **70** | Ampicillin, Piperacillin/tazobactam (I), Cefuroxime axetil, Ceftriaxone,Cefepime, Amikacin (I), Gentamicin (I), Trimethoprim-sulfamethoxazole, Ciprofloxacin |
| **71** | Ampicillin:S, Gentamicin:S, Ciprofloxacin:S, Trimethoprim-sulfamethoxazole:S |
| **72** | Ampicillin:S, Gentamicin:S, Cefuroxime axetil:S, Ciprofloxacin:S, Trimethoprim-sulfamethoxazole:S |
| **73** | Ampicillin |
| **74** | Cefuroxime axetil, Ceftriaxone,Cefuroxime(I), Ampicillin, Piperacillin/tazobactam (I) |
| **75** | ESBL+, Gentmicin, Ciprofloxacin,Trimethoprim-sulfamethoxazole,Cefuroxime, Ceftriaxone,Cefepime, Ampicillin, Piperacillin/tazobactam (I), Cefuroxime axetil |
| **76** | ESBL+,Cefuroxime, Ampicillin, Piperacillin/tazobactam,Cefuroxime axetil, Ceftriaxone,Cefepime (I), Trimethoprim-sulfamethoxazole |
| **79** | Ampicillin:S, Gentamicin:S, Cefuroxime axetil:S, Ciprofloxacin:S, Trimethoprim-sulfamethoxazole:S |
| **80** | ESBL+, Ampicillin, Piperacillin/tazobactam, Cefuroxime axetil, Ceftriaxone,Cefepime,Amikacin (I), Gentamicin, Ciprofloxacin,Trimethoprim-sulfamethoxazole |
| **81** | Amikacin (I) |
| **82** | ESBL+,Cefuroxime, Ampicillin, Piperacillin/tazobactam,Cefuroxime axetil, Ceftriaxone,Cefepime ,Amikacin (I), Gentamicin, Trimethoprim-sulfamethoxazole |
| **83** | ESBL+, Cefepime, Gentamicin, Ciprofloxacin, Trimethoprim-sulfamethoxazole, Ampicillin, Piperacillin/Tazobactam (I), Cefuroxime axetil, Ceftriaxone |
| **85** | ESBL+, Ampicillin,Cefuroxime, Cefuroxime axetil, Ceftriaxone,Cefepime (I), Gentamicin, Ciprofloxacin, Trimethoprim-sulfamethoxazole |
| **87** | ESBL +, Ampicillin , Cefuroxime axetil, Ceftriaxone, Cefepime (I), Amikacin (I), Gentamicin, Ciprofloxacin,Trimethoprim-sulfamethoxazole |
| **88** | Ampicillin, Piperacillin/tazobactam |
| **89** | ESBL+, Cefuroxime, Ampicillin, Piperacillin/tazobactam (I), sefuroksim Cefuroxime axetil, Ceftriaxone,Cefepime, Amikacin (I), gentamicin, Ciprofloxacin,Trimethoprim-sulfamethoxazole |
| **90** | ESBL+,Cefuroxime, Ampicillin, Piperacillin/tazobactam(I), Cefuroxime axetil, Ceftriaxone,Cefepime |
| **91** | Amikacin (I) |
| **92** | Ampicillin:S, Gentamicin:S, Cefuroxime axetil:S, Ciprofloxacin:S, Trimethoprim-sulfamethoxazole:S |
| **93** | Amikacin (I), Ciprofloxacin |
| **94** | ESB+, Cefuroxime,Ampicillin, Piperacillin/tazobactam (I), Cefuroxime axetil,Ceftriaxone,Cefepime, Gentamicin |
| **95** | ESBL+,Cefuroxime,Ampicillin, Piperacillin/tazobactam, Cefuroxime axetil, Ceftriaxone,Cefepime (I) |
| **96** | Ampicillin:S, Gentamicin:S, Cefuroxime axetil:S, Amikacin:S, Trimethoprim-sulfamethoxazole:S |
| **97** | ESBL+,Cefuroxime, Ampicillin, Piperacillin/tazobactam (I), Cefuroxime axetil, Ceftriaxone,Cefepime |
| **98** | Ampicillin:S, Gentamicin:S, Cefuroxime axetil:S, Amikacin:S, Trimethoprim-sulfamethoxazole:S |
| **99** | ESBL+, Cefuroxime axetil,Cefuroxime, Ampicillin,Piperacillin/tazobactam, Cefuroxime axetil, Ceftriaxone (I),Cefepime (I), gentamsin, Ciprofloxacin, Trimethoprim-sulfamethoxazole |
| **100** | ESBL+,Cefuroxime, Ampicillin,Piperacillin/tazobactam (I), Cefuroxime axetil, Ceftriaxone,Cefepime , Amikacin (I), Ciprofloxacin,Trimethoprim-sulfamethoxazole |
| **101** | ESBL+, Cefuroxime axetil, Ceftriaxone,Cefuroxime, Ampicillin,Cefepime, Trimethoprim-sulfamethoxazole |
| **102** | ESBL+,Cefuroxime, Ampicillin, Piperacillin/tazobactam,Cefuroxime axetil,Cefepime, Ceftriaxone, Trimethoprim-sulfamethoxazole |
| **103** | ESBL+,Cefuroxime, Ampicillin, Piperacillin/tazobactam, Ceftriaxone, Cefepime, Cefuroxime axetil, Amikacin (I), Ciprofloxacin,Trimethoprim-sulfamethoxazole |
| **104** | Cefuroxime, Ampicillin,Cefuroxime axetil, Ciprofloxacin, |
| **105** | ESBL+,Cefuroxime, Ampicillin, Piperacillin/tazobactam(I),Cefuroxime axetil,Ceftriaxone,Cefepime,Amikacin (I), Ciprofloxacin |
